# Thermal Stabilization of Nafion with Nanocarbon Materials

**DOI:** 10.3390/polym15092070

**Published:** 2023-04-27

**Authors:** Anna O. Krasnova, Nadezhda V. Glebova, Angelina G. Kastsova, Maxim K. Rabchinskii, Andrey A. Nechitailov

**Affiliations:** Department of Solid State Electronics, Ioffe Institute, 194021 St. Petersburg, Russia

**Keywords:** Nafion polymer degradation, Nafion stabilization, carbon nanotubes, thermally expanded graphite, proton-conducting polymer

## Abstract

The stability of Nafion–carbon composites is important for the efficient functioning of fuel cells. The thermal decomposition of Nafion, nanostructured carbon materials, such as multi-walled carbon nanotubes, graphene-like materials, and their composites, have been studied using constant heating rate thermogravimetry in air. Materials were characterized by quantitative and qualitative analysis methods, such as thermogravimetry, X-ray photoelectron spectroscopy, scanning, and transmission electron microscopy with field emission. In Nafion–carbon composites, an increase in the thermal stability of the Nafion polymer is observed due to the formation of surface compounds at the Nafion–carbon interface. In this case, the degree of stabilization is affected by both the component composition of the composite and the structure of the nanocarbon material. The greatest effect was obtained in the case of using thermally expanded graphite (few-layer graphene). Nafion is distributed to a greater extent over the surface of the carbon material due to its high structural accessibility. The most thermally stable composite is Nafion–graphene in a mass ratio of components 1:4 with one stage Nafion degradation at 422 °C, whereas the degradation of pristine Nafion occurs in three stages at 341, 413, and 430 °C. The dependences of thermal stability and features of thermal degradation on the composition and structure of composites are discussed.

## 1. Introduction

The proton-exchange membrane fuel cell (PEM FC) is an important technology for renewable energy. Hydrogen gas, which is the main source of fuel in FCs, is relatively readily available, and FC exhaust gases do not contain greenhouse gases, unlike fossil fuel-based energy sources.

Many recent studies have addressed the aging and reliability of membrane and electrodes of PEM FCs. This has resulted in some understanding of their degradation mechanisms and of measures to counteract degradation in order to increase their durability and reduce their price [1,2,3,4,5,6,7,8,9,10].

A study of membrane degradation in [6] showed that membrane reinforcement slows down its degradation. Glebova et al. [9] studied the mass transport in the electrodes during their aging and suggested that Nafion is washed out during the aging process. Menshchikov et al. [7] studied the stability of electrocatalysts based on platinum–copper alloys and showed their high resistance to electrochemical attack.

Degradation of the dense-conducting polymer is one of the most vulnerable processes since measures to counteract them are difficult at the moment.

Although there are numerous publications on the mechanisms of membrane degradation, only a small percentage is devoted to the degradation of the ionomer binder in the electrodes. This is due to certain difficulties of such studies, such as small amounts in the electrodes or close mixing with other components, which actually leads to distortion of the ionomer structure, etc.

Danerol et al. [11] showed X-ray diffraction results indicating structural changes in the ionomer during aging. This structural change is due to a possible reduction in the number of side chains as a result of chemical degradation or the formation of shorter ionomer chains. Kaddouri [12] showed that the chain organization (structure) of the ionomer binder changed after the FC operation (in different ways at the cathode and anode). Accelerated stress tests revealed structural changes and loss of material after aging [13,14,15]. In [14], the electrodes were analyzed after voltage cycling in pure hydrogen and hydrogen with 5 ppm CO at the anode: X-ray photoelectron spectroscopy (XPS) analysis revealed a decrease in CF_2_- and CF_3_-bonds corresponding to an ionomer loss of approximately 13.2% after 550 h of stress cycling with pure hydrogen and approximately 10.6% after 630 h of stress cycling with hydrogen contaminated with 5 ppm CO. Zhang et al. [13] confirmed the loss of ionomer when the fluorine content was reduced from 50% to 39% after 300 h of voltage cycling.

Detection of ionomer decomposition is a nontrivial task since express analysis of polymer structure is difficult to implement. Many studies use the detection of fluorine emitted during the decomposition of the ionomer as an indicator of Nafion degradation.

Today, a number of measures are used to stabilize the ionomer in mixed conductivity electrodes and PEMs, which mainly entail the introduction of components that actively destroy free radicals.

A powerful strategy to reduce chemical degradation of the membrane is to introduce radical scavengers, which can deactivate harmful free radicals before they react with the polymer chains. Several inorganic compounds have been proposed in this regard: metal oxides, such as titanium dioxide (TiO_2_), zirconium dioxide (ZrO_2_), tin dioxide (SnO_2_), manganese dioxide (MnO_2_), or cerium dioxide (CeO_2_); metal particles, such as Ce, Mn, Pd, Pt, Ag, or Au [16]; and calcium carbonate (CaCO_3_) from eggshells [17,18]. Among them, cerium (Ce) and manganese (Mn) derivatives (ionic forms [19,20], metal oxides [21,22,23,24,25], metal nanoparticles [26], or particles immobilized on a silica carrier [27]) are most commonly used in PEM FCs. Evidently, the use of metals that can yield cations is associated with the danger of losing the proton-conducting properties of the ionomer due to the ion exchange of protons for metal ions. Another strategy to increase structural organization of Nafion is utilizing anionic surfactants as reposted in [28,29].

The thermal stability and durability of PEM FCs are studied in works [30,31,32,33] that use and investigate the method of creating filled composite membranes with various fillers that act as stabilizers.

Glebova et al. [8] reported on a slightly different approach to stabilize Nafion. It was shown that the thermal stability of the proton-conducting polymer Nafion on the graphene surface increased in comparison with the bulk polymer.

Therefore, it should be noted at this point that, like the catalyst (Pt on a carbon carrier (Pt/C)), the bonding ionomer in the catalyst layers is also subject to degradation. Nevertheless, the degradation of the ionomer binder is less studied than the degradation of Pt particles or carbon-based carriers. This is due to the low content of the ionomer in the catalytic layers and the difficulty of separating the degradation of the ionomer membrane from the degradation of the ionomer binder during FC operation. Accordingly, methods to combat the degradation of the proton-conducting polymer included in the electrode are insufficiently developed.

Today, the use of various forms of nanostructured carbon, such as carbon nanotubes (CNTs), fullerenes, graphene, etc., in electrochemical electrodes is an established trend [34,35,36]. Each form of carbon is characterized by certain functional properties, which are used when the composite is made [37,38,39,40].

One of the most important properties of electrode materials is their thermal stability. This parameter is important both at the stage of production (some technological processes are carried out at high temperatures) and in the use of final products. Proton-conducting polymer is the most thermally unstable component.

Electrodes containing the proton-conducting polymer Nafion are used at relatively low temperatures, usually up to 80 °C [37]. This is related to the processes of thermal degradation (at high temperatures, the electrode material is destroyed, causing it to lose its functional properties: proton, electronic conductivity, etc.). The temperature threshold at which the components are destroyed is at relatively low levels, so it is of high importance to increase it, thus increasing the thermal stability. Thermal stability is important both in the operation of the electrodes and in their production, which involves some stages during heating, such as thermal compression [41]. Nafion is the most thermally unstable component of electrode. Its reversible dehydration already occurs at approximately *T* = 100 °C. As the temperature further increases, irreversible decomposition of the polymer takes place through a series of successive stages: desulphation, detachment of side chains of atoms, and destruction of the main skeleton of the molecule [8,31]. It was shown in study [31] that thermal decomposition is initiated by the detachment of the side chain at temperatures above 350 °C, resulting in carbonyls remaining in the main chain in the side chain locations.

Perfluoroalkanes are released at temperatures greater than 400 °C as a result of breaking the main chain, and their further decomposition products prevail at temperatures greater than 500 °C. Matos et al. [31] analyzed the effect of TiO_2_ additive on the thermal properties of Nafion and showed an increase in the thermal stability of Nafion. De Volder et al. [42] showed a high stabilizing effect of graphene plates on Nafion-containing composite materials. The present work is a development of this direction and is devoted to the study of composites containing CNTs and few-layer graphene, obtained in the process of ultrasonic dispersion of thermally expanded graphite (TEG).

The objective of this work is to study the thermal stability and degradation features of Nafion in composites containing CNTs and few-layer graphene, to study the mechanisms of ionomer stabilization by different carbon nanostructured materials (CNM), and to search for ways to increase its stability.

## 2. Materials and Methods

### 2.1. The Following Materials Were Used in the Experiments

Multi-walled CNTs of the Taunit MD brand (NanoTechCenter LLC, Tambov, Russia), was purified in HNO_3_ (70.00%, 18-4, Component-Reactiv LLC, Moscow, Russia). TEG was obtained from NPO “Graphene Materials” LLC, Russia using the technology described in [43]. E-TEK platinized carbon black (40% Pt) [44], Nafion solution DE2020 (DuPont™, Wilmington, DE, USA), sopropanol (99.80%, ECOS-1 JSC), and deionized water with resistivity at room temperature ρ ≥ 18 MOhm × cm were used.

### 2.2. Differential Thermal Studies

The study of thermal degradation was carried out on a derivatograph of Mettler-Toledo TGA/DSC 1 type with the software STARe System (Mettler-Toledo LLC, Columbus, OH, USA) with blowing air through the derivatograph chamber at a rate of 30 cm^3^/min in the mode of uniform temperature rise at a rate of 10 K/min in the temperature range *T* = 35–1000 °C. A sample of material weighing a few mg was placed in an alund crucible, and mass (thermogravimetric (TG)) and thermal (differential thermal (DT)) curves were recorded during heating.

In order to study Nafion, it was prepared in water–isopropanol solution in the volume ratio isopropanol:water = 1:1 (with Nafion content of 2%, the solution was dried on the glass at a.c. to air-dry state (relative humidity ~40–50%), and then the dry residue was removed with a metal (stainless steel) spatula.

Multicomponent systems were prepared by mechanical mixing and subsequent ultrasonic homogenization of the initial components. For this purpose, precise proportions of materials were mixed in a polyethylene test tube, and the mixture of isopropanol and water, taken in the volume ratio of 1:1, was added. The solid–liquid ratio in the dispersion was maintained in the range 1:40–1:80, depending on the content of the carbon component. The higher its content, the more liquid phase was added to ensure complete wetting of the solid components. The test tube was placed in a Branson 3510 ultrasound bath (Branson Ultrasonics Corporation, Danbury, CT, USA). Treatment in an ultrasonic bath was carried out at an operating frequency of *f* = 40 kHz and a power of 130 W for ~30–50 h until a visually uniform dispersion was obtained. The dispersion was dried on glass at a.c. to air-dry (~40–50% relative humidity), and then the dry residue was removed with a metal (stainless steel) spatula. Before thermogravimetric analysis, the prepared samples were additionally dried at *T* = 100 °C for 10 min.

### 2.3. Microscopic Studies

Target-oriented approach was utilized for the optimization of the analytic measurements [45]. Before measurements, the samples were mounted on a 3 mm copper grid and fixed in a grid holder. Samples morphology was studied using Hitachi SU8000 field-emission scanning electron microscope (FE-SEM). Images were acquired in bright-field STEM mode at 30 kV accelerating voltage.

### 2.4. XPS

Chemical analysis of Nafion and composite samples, Nafion:TEG, was performed by the means of XPS. The spectra were collected using the NanoPES experimental station at the Kurchatov Synchrotron Radiation Source (National Research Center Kurchatov Institute, Moscow, Russia). XPS system equipped with a XR-MF microfocus source (Al Kα, *hν* = 1486.61 eV) and Phoibos150 analyzer was used. The operating pressure in the sampling chamber was below 10^−9^ Torr. The spectra were calibrated with respect to the Au 4f7/2 line (84.0 eV). Samples were aligned in the beam by maximizing photoelectron counts corresponding to the primary C 1s peak in C–C bonds located at a binding energy of 284.7 eV. XPS survey spectra were measured with the energy step of 0.5 eV, while for the S 2p spectra, this value was 0.05 eV. Atomic concentrations of different elements were calculated on the basis of the photoelectron intensities of each element and the elemental sensitivity factors provided by PHI. Samples were scanned at different locations, and the peak intensity and composition at different locations were compared to assure uniformity of film composition over the sample surface. CasaXPS software (Version 2.3.16Dev52, Casa Software Ltd., Teignmouth, UK) was applied for the deconvolution of the acquired S 2p X-ray photoelectron spectra. All the spectra were fitted with a Shirley background.

### 2.5. Calculation and Processing of Results

Experimental data processing in the study was carried out by means of derivatograph software. To calculate the specific thermal effect of Nafion oxidation, numerical integration of the area bounded by DT and the baseline according to relation (1) was used:

(1) $$\Delta H_{sp}=(\int_{m_{1}}^{m_{2}}\left(\left(dH/d\tau \right)\times d\tau \right)/\left(m_{1}-m_{2}\right)$$

where Δ*H_sp_*—specific heat of oxidation, kJ/g; *τ*—time, s; and *m*_1_ and *m*_2_—initial and final mass of the sample, respectively.

## 3. Results and Discussion

**Figure 1:**
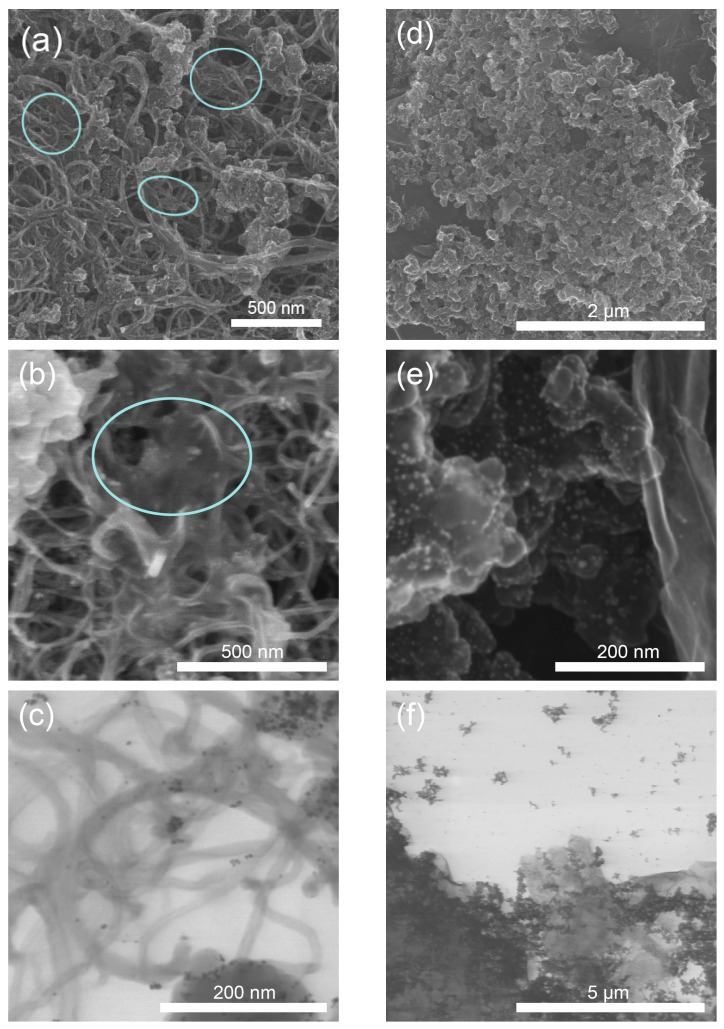
Microscopic images of Nafion:CNTs:Pt/C (1:0.75:0.75): (**a**) and (**b**) SEM; (**c**) TEM. Nafion:TEG:Pt/C (1:0.75:0.75): (**d**) and (**e**) SEM; (**f**) TEM. The circles indicate the Nafion polymer on the CNM surface.

Figure 1 shows typical micrographs of three-component composites containing CNTs and TEG as functional additives.

Spherical globules with light (SEM) and dark (TEM) dots represent carbon soot with platinum nanoparticles on it. As can be seen from the figure, the CNTs composite contains long CNTs that are longer than a micron in length at ~20 nm thickness. The tubes are twisted together to form something similar in structure to a tangle, containing voids of hundreds of nm in size (Figure 1a–c). The SEM images of the composite with CNTs (Figure 1a,b) show shapeless areas of Nafion on the surface (some areas are marked in Figure 1a,b). They are represented by areas with indistinct boundaries. It can be seen that Nafion is mostly on the surface of the material, with a high proportion of the polymer not directly in contact with the CNT surface. The sample with TEG (Figure 1d–f) contains micron-sized graphene plates. The image (Figure 1e) shows a curved sheet of few-layer graphene whose thickness can be estimated as ~15 nm. Study [46] showed that the ultrasonic dispersion of TEG in the presence of Nafion causes the splitting of graphite layers with the formation of thinner structures up to few-layer (2–3 layers) graphene. In this case, there is adsorption of Nafion on the surface of graphene plates with a high degree of contact of these materials. In Figure 1f, one can see the surface of a graphene plate with an area of a few μm^2^, with the Pt/C catalyst located on it. According to the contrast (degree of transparency), it is possible to make an estimate of graphene plate thickness, which is significantly less than that of the Pt/C globules.

**Figure 2:**
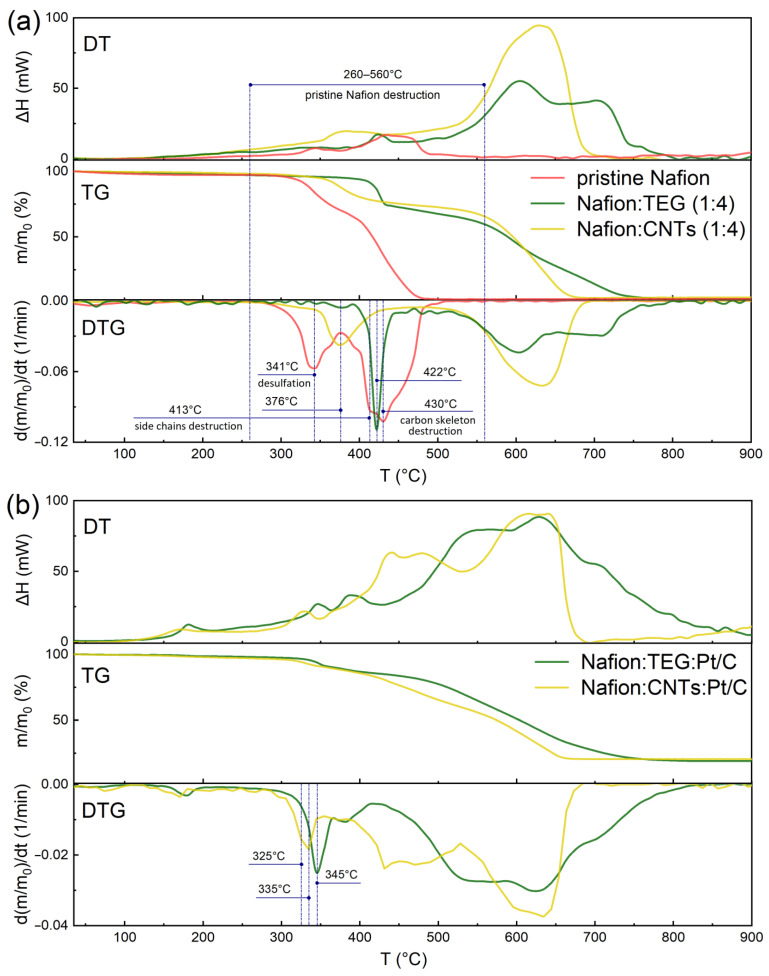
Derivatograms of composite materials based on proton-conducting polymer Nafion (wt.:wt.:wt.): (**a**) Nafion:CNM:(w/o Pt) (1:4:0) and (**b**) Nafion:CNM:Pt/C (1:4.5:4.5). The heating rate is 10 K/min.

The process of thermal degradation of Nafion (Figure 2a) occurs in three stages, which correspond to three peaks on the DTG curve. The derivatograms indicate a low-temperature peak (341 °C), which is associated with desulfation, and two peaks at higher temperatures (413 °C and 430 °C), at which the destruction of the side chains and the carbon skeleton occurs [47,48,49]. However, as can be seen from the figure, the second and third peaks strongly overlap and are barely resolvable. This is characterized on the TG-curve by the presence of two stages of mass loss with a different slope. The complete thermal oxidation of the proton-conducting polymer Nafion (Figure 2a) occurs in the temperature range 260–560 °C. Oxidative degradation is accompanied by corresponding exothermic effects, which are clearly visible on the DT curve.

The addition of CNTs at a mass ratio of Nafion:CNTs = 1:4 (Figure 2a) leads to a shift of the destruction temperature to a higher temperature region, combining the individual destruction stages into one with a maximum rate at 376 °C and narrowing the destruction temperature interval. The addition of platinized carbon soot to the composite (Figure 2b) results in a decrease in the temperature of the maximum rate of degradation to 335 °C, while the process is also characterized by a single peak in the DTG curve. In this case, the lowering of the destruction temperature is likely due to the catalytic effect of platinum. Qualitatively similar patterns are observed for composites containing TEG. At the same time, the stabilizing effect of TEG is much stronger.

The higher-temperature mass loss processes in the derivatograms (Figure 2) are associated with the oxidation of the corresponding carbon materials (CNTs, TEG, and carbon soot). The permanent residue for platinum-containing composites corresponds to the remaining platinum. Table 1 summarizes the numerical values of degradation temperatures for the examined composites.

Several conclusions emerge from the table. The presence of CNTs and TEG increases the thermal stability of Nafion, while the presence of platinum decreases it. In this case, the mechanism of Nafion degradation changes. In the case of the pristine polymer, a multistage process is observed, while in the case of composites containing carbon materials (CNTs, TEG, and carbon black) and platinum, the temperatures of the individual stages converge, which is ultimately expressed by one step on the TG and one peak on the DTG curves. CNTs and TEG and their proportion in the composite have different effects on the processes described above. Thus, the composite of Nafion:CNTs (1:1) still shows two peaks of Nafion degradation on the DTG curve. When the CNTs fraction is increased to 80% (Nafion:CNTs, 1:4), the peaks merge into one and do not resolve under the experimental conditions. TEG has a significantly greater effect on the thermal stability of Nafion compared with CNTs. With 50% TEG content (Nafion:TEG, 1:1), degradation already occurs in one step, and the temperatures of maximum mass loss rates are shifted more toward the high-temperature region. Therefore, we can conclude that the specific contact area of Nafion with the carbon material in the composite plays a decisive role. Figure 1 shows that the spatial accessibility of CNTs to the polymer is limited by their structure. A considerable part of the Nafion is not in contact with the CNT surface when in a bulk state. Meanwhile, the TEG plates are spatially open and more accessible to the polymer.

### 3.1. Nafion Destruction Thermal Effects

The thermal effects of Nafion degradation and XPS spectra for pristine Nafion and its composite with Nafion:TEG (which has the most pronounced stabilization effect) were examined to confirm the assumption that Nafion contact with the carbon material surface plays a role in polymer stabilization.

The thermal effects of Nafion degradation were studied, and they should change with respect to pristine Nafion in the case of polymer adsorption on the surface of carbon material, with the possible formation of surface compounds.

Table 2 shows quantitative data for some composites of different formulations. The degradation occurs in all cases with an exothermic effect. Complete degradation of the pristine (bulk) polymer (Figure 2a, Table 2) occurs with a specific heat equal to 2.14 kJ/g. Composites containing CNTs have a slightly higher thermal effect and are approximately 3 kJ/g (Table 2). Composites containing relatively large amounts of TEG (Nafion:TEG, 1:1) have a thermal effect value (2.24 kJ/g) close to that of pristine Nafion (2.14 kJ/g). A significant decrease in the specific heat of degradation (1.54 kJ/g) is observed when the polymer fraction (Nafion:TEG, 1:4) is reduced.

Composites with a large fraction of Nafion have a significant portion of it in the bulk state, which means that it is not in direct contact with the surface of the carbon material, and, accordingly, its degradation occurs with a specific heat effect close to that of pristine Nafion, which is observed for the sample composition of Nafion:TEG, 1:1. When the fraction of Nafion is reduced to 20% (Nafion:CNTs, 1:4), the fraction of surface Nafion (the polymer directly in contact with the TEG surface) increases, while the fraction of bulk Nafion decreases. This leads to a decrease in the specific heat of polymer degradation in the composite.

The excess of the specific thermal effect of Nafion degradation for composites with CNTs can be explained by a significant contribution of oxidation of the carbon material. Figure 2 clearly shows that the DTG curves are located in the negative region. That is, there is a background oxidation of the carbon material. The background oxidation of the carbon material is accompanied by a corresponding exothermic effect, which is characterized by the positive slope of the DT curves of the composites (Figure 2) in contrast to pristine Nafion. Given the significantly higher value of the specific heat of combustion of graphite (~32 kJ/g [50]) compared with the specific heat of Nafion degradation (~2 kJ/g (Table 2)), the contribution from the background oxidation of the carbon material to the total thermal effect can be significant. One can say that there are two competing, oppositely directed factors in the total heat effect: a decrease in the heat of Nafion degradation as a result of surface stabilization on the carbon material and an increase in the heat effect due to background oxidation of the carbon material. The stabilizing effect of TEG is higher than CNTs.

It should be noted that the components in the composite are found in at least two structural states: in contact with each other (surface material) and in the bulk (bulk material) state. When one component has a relatively large fraction, the ratio of surface and bulk quantities is small, and vice versa. The 1:4 ratio of CNTs in the Nafion:CNTs sample has a high proportion of surface Nafion, and the effect of CNTs is stronger than in the 1:1 ratio of Nafion:CNTs, which have a higher proportion of bulk Nafion.

The stabilizing effect of TEG is related to the structural features of the materials. Due to the fibrous structure with a large number of pores, CNTs contribute to the structuring of Nafion in the composite mainly in the form of agglomerates since they have a pore structure difficult to access for uniform penetration of the polymer [10], while TEG plates are more spatially accessible to Nafion, and the proportion of surface polymer is higher ceteris paribus.

Thus, the data obtained to study the thermal effects of Nafion degradation in composites of different formulations are consistent with the ideas about the influence of the contact surface of Nafion with the carbon material and the formation of stabilizing surface compounds.

### 3.2. XPS Study

**Figure 3:**
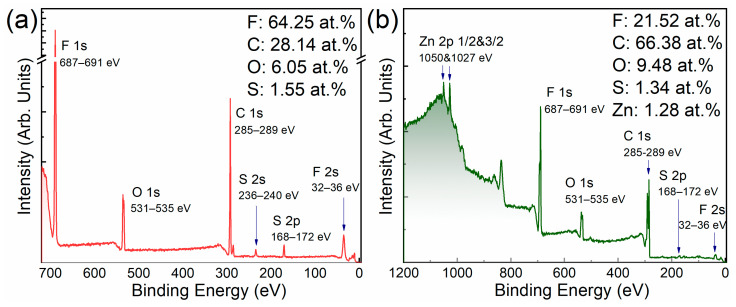
Survey XPS spectra of the materials: (**a**) pristine Nafion and (**b**) Nafion:TEG (1:4) composite.

The materials were examined by the XPS method in order to establish the fact of interaction of the Nafion polymer with the graphene surface. Figure 3 shows the overview spectra of pristine Nafion and its composite with Nafion:TEG, 1:4.

**Figure 4:**
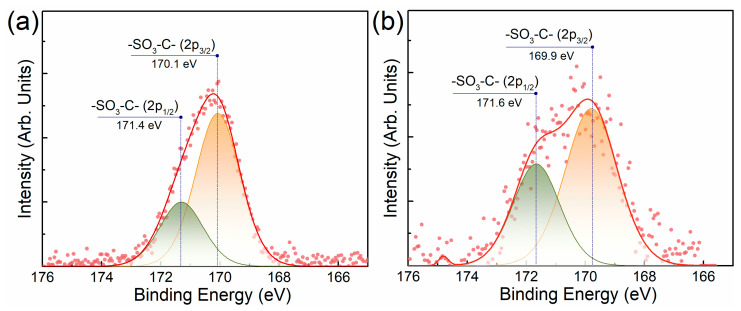
S 2p XPS spectra: (**a**) pristine Nafion and (**b**) Nafion:TEG (1:4) composite.

Figure 4 shows that the S 2p electrons have a binding energy of approximately 170 eV. The figure shows that there is a spin–orbit splitting of the spectrum. The ratio between the intensities of the doublet peaks is different for pristine Nafion and for the composite. The composite has a more degraded energy level. As can be seen from the figure, the values of the electron binding energies for Nafion and the composite differ by 0.2 eV.

This difference in the XPS spectra of pristine Nafion and composite indicates the presence of interaction between Nafion and the surface of the graphene plate in TEG. Thus, the XPS spectra confirm the presence of the interaction of Nafion with the surface of graphene plates, which is consistent with the thermogravimetric data on the thermal stabilization of the polymer.

Composite materials of the same quality composition with low Nafion content are more thermally stable than materials with high Nafion content. Materials containing TEG as a carbon component are more thermally stable than CNTs composites. The presence of CNTs in the composite stabilizes the material less than TEG.

These conclusions can be explained by the peculiarities of structure formation in composites with different carbon materials. The used carbon materials have fundamentally different structures: TEG in the composite transforms into few-layer graphene, whose plate surface is spatially accessible to Nafion in the composite, while the structural elements of CNTs are long twisted tubes with a fibrous structure that has hard-to-reach pores for Nafion molecules. As a result, the specific surface area of Nafion contact with few-layer graphene is higher than with CNTs, the fraction of surface Nafion is greater than bulk Nafion, and the effect of thermal stabilization is more pronounced.

The experimental patterns are consistent with the concept that takes into account the existence of a surface and bulk Nafion. Samples with a higher proportion of surface polymer are more thermally stable. The sample with TEG containing 20% Nafion (Nafion:TEG, 1:4) is more thermally stable than the sample with the same Nafion content (20%), but with CNTs, due to different structural organization, CNTs are less covered by the polymer because they have voids that prevent polymer penetration.

## 4. Conclusions

The mechanism of Nafion thermal degradation changes in the presence of the studied carbon materials (multi-walled CNTs, few-layer graphene, and carbon soot): the temperatures of individual stages of the multi-stage process converge, resulting in polymer degradation in one stage, which has one characteristic temperature—at its maximum rate.

The presence of nanostructured carbon materials, such as multi-walled CNTs and few-layer graphene, increases the thermal stability of Nafion. The strongest stabilizing effect is observed in Nafion–graphene composite (mass ratio of components—1:4) with one stage Nafion degradation at 422 °C, whereas the degradation of pristine Nafion occurs in three stages at 341, 413, and 430 °C.

The increase in stability is due to the interaction of the polymer with the surface of the carbon material, resulting in a reduction in the energy of the system. This is expressed in a decrease in the specific heat of Nafion degradation in the composite compared with the pristine polymer. The key factor determining the degree of Nafion stabilization in the composite is the ratio of its surface and bulk states. The more of the polymer that is in the surface state, the higher its degree of stabilization. The structure of the carbon material plays an important role in stabilizing Nafion in the composite. The high surface availability of carbon nanofragments and its large specific surface area increase the efficiency of Nafion stabilization through the formation of its adsorbates. Among the studied carbon materials, the most stabilizing effect was exerted by the few-layer graphene.

## Figures and Tables

**Table 1:** Nafion degradation temperatures (DTG Nafion thermal degradation peaks), °C at a heating rate of 10 K/min.

Composite Content (wt.:wt.)	*T*, °C	Note
Nafion (Figure 2a)	341, 413, 430	three stages of degradation
Nafion:CNTs, 1:1	348, 409	two stages of degradation
Nafion:CNTs, 1:4 (Figure 2a)	376	degradation combined in one stage
Nafion:TEG, 1:1	353	degradation combined in one stage
Nafion:TEG, 1:4 (Figure 2a)	422	degradation combined in one stage
Nafion:Pt/C, 1:1.5	304	degradation combined in one stage
Nafion:CNTs:Pt/C, 1:4.5:4.5 (Figure 2b)	325, 335	two stages of degradation
Nafion:TEG:Pt/C, 1:4.5:4.5 (Figure 2b)	345	degradation combined in one stage

**Table 2:** Specific thermal effects Δ*H* of Nafion degradation in composites with nanostructured carbon materials.

Nafion Composite: CNM (wt.: wt.)	Combustion Interval, °C	Combustion Interval Width, Δ*T*, °C	Δ*H*, kJ/g
Nafion	260–560	300	2.14
Nafion:CNTs, 1:1	330–430	100	3.15
Nafion:CNTs, 1:4	290–450	160	3.30
Nafion:TEG, 1:1	430–460	130	2.24
Nafion:TEG, 1:4	380–440	60	1.54

## Data Availability

The data presented in this study are available on request from the corresponding author.
